# Prognostic Role of Inflammatory Biomarkers in Gastrointestinal Neuroendocrine Neoplasms: A Cross‐Sectional Study

**DOI:** 10.1002/hsr2.71619

**Published:** 2025-12-28

**Authors:** Hoda Mahdavi, Vahid Kaveh, Mahdiye Ijadi Nasrabadi, Mohammad Amin Karimi, Mehdi Azizmohammad Looha

**Affiliations:** ^1^ Radiation Oncology Department, School of Medicine Iran University of Medical Sciences Tehran Iran; ^2^ Hematology and Oncology Department, School of Medicine Iran University of Medical Sciences Tehran Iran; ^3^ Neuromusculoskeletal Research Center, Department of Physical Medicine and Rehabilitation, School of Medicine Iran University of Medical Sciences Tehran Iran; ^4^ School of Medicine Shahid Beheshti University of Medical Sciences Tehran Iran; ^5^ Basic and Molecular Epidemiology of Gastrointestinal Disorders Research Center, Research Institute for Gastroenterology and Liver Diseases Shahid Beheshti University of Medical Sciences Tehran Iran

**Keywords:** gastrointestinal neuroendocrine neoplasms, Ki‐67, neutrophil‐to‐lymphocyte ratio, platelet‐to‐lymphocyte ratio, prognostic biomarkers

## Abstract

**Background and Aims:**

Gastrointestinal neuroendocrine neoplasms (g‐NENs) represent a heterogeneous group of tumors with variable prognoses. Inflammation‐based markers, such as the neutrophil‐to‐lymphocyte ratio (NLR) and platelet‐to‐lymphocyte ratio (PLR), have emerged as potential prognostic indicators in various malignancies. This study aimed to evaluate the prognostic significance of NLR and PLR in g‐NENs and their associations with histopathological features and survival outcomes.

**Methods:**

A retrospective, cross‐sectional study was conducted on 80 patients with histologically confirmed g‐NENs from April 2017 to March 2019. Baseline NLR and PLR were calculated, and their relationships with tumor grade, Ki‐67 proliferation index, metastatic status, and survival outcomes were analyzed. Cox regression and logistic regression models were used to determine independent prognostic factors.

**Results:**

The hazard of death was approximately one‐third in the low NLR group after adjusting for metastasis and tumor grade (HR = 0.33, 95% CI: 0.14–0.80, *p* < 0.05). Elevated PLR was associated with poor outcomes; however, the difference between the high and low PLR groups was not statistically significant. Higher tumor grades and elevated Ki‐67 indices correlated with worse survival and increased metastatic potential (*p* < 0.001). No significant associations were observed for CRP or ESR levels with survival.

**Conclusion:**

Our findings suggest that NLR may serve as an independent and accessible prognostic marker in g‐NENs, potentially offering a cost‐effective tool to support clinical decision‐making. The combined assessment of inflammatory biomarkers and histopathological parameters could improve prognostic accuracy and help guide personalized management strategies in patients with g‐NENs. Nevertheless, these observations should be interpreted with caution due to the retrospective, single‐center design and limited sample size. Future large‐scale, prospective studies are warranted to confirm and expand upon these results.

## 1 Introduction

Neuroendocrine (NE) neoplasms comprise a heterogeneous group of tumors ranging from indolent to highly aggressive, with an estimated prevalence of 2–8 per 100,000 individuals. The gastrointestinal (GI) tract is the most commonly affected site, accounting for 55%–70% of all NE tumors [1]. Gastroenteropancreatic neuroendocrine neoplasms (GEP‐NENs) represent nearly two‐thirds of all neuroendocrine neoplasms (NENs) [2, 3, 4], and their incidence has risen over recent decades, likely due to improved diagnostics and greater clinical awareness. Despite this progress, their biological diversity continues to challenge diagnosis, classification, and management [5, 6].

Recent clinical evidence highlights the expanding therapeutic landscape for advanced and metastatic NENs. Systemic options such as somatostatin analogs, everolimus, sunitinib, and peptide receptor radionuclide therapy (PRRT) have demonstrated significant improvements in progression‐free survival and disease control in well‐differentiated tumors. Nevertheless, chemotherapy remains a cornerstone of management, particularly for pancreatic or high‐grade, rapidly progressive NENs, where it continues to provide meaningful tumor shrinkage and symptomatic relief [7, 8, 9].

Despite improvements in detection, the rarity of GEP‐NENs means that reliable prognostic markers are often lacking, and many cases are diagnosed at advanced stages, contributing to poor outcomes [5, 6, 10, 11]. Conventional prognostic indicators, such as tumor grade and histopathological findings, often require surgical specimens, limiting their utility in nonsurgical cases. Furthermore, genetic variability within these tumors complicates the development of targeted therapies and adds to the difficulty of predicting disease progression [5, 6]. The prognosis of gastroenteropancreatic neuroendocrine neoplasms (g‐NENs) is influenced by factors such as tumor stage, histological features, and adherence to standardized management protocols, which are critical for accurate classification [12]. The World Health Organization (WHO) grading system incorporates key parameters, including the Ki‐67 proliferation index and mitotic count (per 2 mm²), to stratify these tumors. Tumors with a mitotic rate under two or a Ki‐67 index ≤ 2% are classified as grade 1 (G1), whereas those with a mitotic rate exceeding 20 or a Ki‐67 index > 20% fall under grade 3 (G3). Grade 2 (G2) tumors lie between these thresholds, while poorly differentiated tumors with high mitotic activity and Ki‐67 levels are categorized as neuroendocrine carcinomas (NECs) [13]. The Ki‐67 labeling index, a nuclear marker linked to cell proliferation, has been integral to the WHO classification of NENs since 2004. Higher Ki‐67 levels are strongly associated with aggressive tumor behavior and worse patient outcomes, making it a critical prognostic marker in the clinical evaluation and management of NENs [14].

Several studies have explored the prognostic significance of various baseline blood parameters in patients with abnormalities, including those in white blood cell count, hemoglobin levels, lactate dehydrogenase (LDH), and C‐reactive protein (CRP) [15, 16]. The neutrophil‐to‐lymphocyte ratio (NLR), an inflammatory biomarker that reflects the balance between innate and adaptive immunity, has been identified as a predictor of overall and disease‐free survival in patients with advanced gastric neuroendocrine neoplasms (g‐NENs). Similarly, in pancreatic g‐NENs, elevated neutrophil‐to‐lymphocyte ratio (NLR) and platelet‐to‐lymphocyte ratio (PLR) were associated with disease progression [17, 18]. In line with this, Cavalcoli et al. demonstrated that low‐dose aspirin use was associated with a protective trend toward improved progression‐free survival, particularly in small‐intestinal NENs, further supporting the concept that systemic inflammation and its modulation can influence tumor progression and prognosis [19].

The NLR and platelet‐to‐lymphocyte ratio (PLR) are derived from routine complete blood counts, offering cost‐effective, widely accessible prognostic tools compared to specialized biomarkers like CRP or ESR. Their calculation requires no additional testing, making them ideal for retrospective or resource‐limited settings.

Despite these findings, our understanding of the prognostic value of cell count‐related indicators in g‐NENs remains limited due to the intrinsic heterogeneity and rarity of these tumors. There is a growing need to investigate the role of pathological and laboratory parameters in predicting the outcomes of patients with g‐NENs across diverse populations. Given the significance of inflammatory responses and immunological interactions in cancer progression, simple blood‐derived measures, such as neutrophil counts, may serve as accessible prognostic tools to guide patient management.

In this study, we aim to evaluate the relationship between histopathological findings, laboratory parameters, and survival outcomes in a cohort of patients with g‐NENs. By elucidating these associations, we hope to contribute to the development of improved prognostic criteria and personalized management strategies for this unique and challenging group of tumors.

## 2 Materials and Methods

### 2.1 Study Design

This study is a cross‐sectional observational study conducted to examine patients with g‐NEN. The goal is to explore the relationship between histopathological and laboratory findings and survival outcomes in these patients. Specifically, the study aims to assess the correlation between biomarkers and disease progression, overall survival, and disease‐free survival, to improve patient management and prognosis. The study adhered to the ethical standards set by the hospital's ethics committee for human experimentation. The research protocol was reviewed and approved by the institutional review board. Written informed consent was obtained for the use of patient data from both paper and electronic medical records.

### 2.2 Setting

This study was conducted at Firoozgar Hospital, a tertiary referral center with a specialized oncology department. The study period covered 2 years, from April 2017 to March 2019. Patients were included if they were referred to the hospital for diagnostic and therapeutic indications related to g‐NEN.

Cause of death was inferred using metastatic status and tumor grade, supported by previous studies showing that metastatic progression is the predominant cause of cancer‐specific mortality in neuroendocrine neoplasms [20, 21, 22].

### 2.3 Participants

Eligibility criteria

- Inclusion criteria: Patients with documented g‐NEN, confirmed by biopsy or surgical specimen via morphological and immunohistochemical examinations, and recorded in the hospital's pathology department.
- Exclusion criteria: Patients with incomplete data regarding NEN classification or those without a blood test at the first visit after diagnosis.

In this study, tumors were classified according to the WHO 2019 criteria into well‐differentiated neuroendocrine tumors (NETs; grades 1–3 based on mitotic count and Ki‐67 index) and poorly differentiated neuroendocrine carcinomas (NECs; small‐cell and large‐cell types) [23].

A simple nonrandom sampling method was employed. Of the 83,966 digital pathology records available in the Hospital Information System (HIS) during the study period, those patients with diagnoses of benign or malignant tissue were initially identified. The search term “neuroendocrine” yielded 242 records. After filtering for gastrointestinal involvement, 161 records were selected. Upon review, duplicates were removed, and the necessary data were extracted, resulting in 80 patients being included in the final analysis.

The study focused on g‐NEN patients specifically diagnosed within the digestive tract. Relevant patient data were extracted from electronic medical records, oncology clinic paper files, and other hospital sources, including radiology and endoscopy records.

To enhance transparency in cohort derivation, each step of the case selection process has now been clearly described, including the initial record search, screening criteria, and reasons for exclusion, to ensure full reproducibility and compliance with STROBE recommendations.

### 2.4 Variables

The key variables in the study included:

- Exposures: Tumor classification (NET or NEC), tumor location (foregut, midgut, hindgut), blood test results (e.g., WBC, lymphocyte count, platelet count).
- Outcomes: Survival status, time to death from any cause.
- Covariates: Age, gender, family history of cancer, tumor grade, and treatment.

NLR and PLR as exposures were calculated from blood test results by dividing neutrophil count and platelet count by lymphocyte count, respectively.

Potential confounders, including age, metastasis status, and tumor grade, were identified a priori based on existing evidence and were adjusted for in multivariable Cox and logistic regression models. Residual confounding due to unmeasured variables such as nutritional status or metabolic comorbidities is acknowledged in the Section 4.

### 2.5 Data Source/Measurements

Data were collected using hospital electronic records, which included demographic information such as age and gender, clinical data including the date of diagnosis and family history of cancer, pathology data detailing tumor location, grade, and classification (NET vs. NEC) according to the WHO 2019 classification, immunohistochemistry results such as Ki‐67, Chromogranin A, and Synaptophysin, and blood test results including WBC, lymphocyte count, hemoglobin (Hb), platelet count, ESR, and CRP. Additionally, survival status was recorded, with death dates obtained from the National Civil Registry and family contacts.

### 2.6 Bias

To mitigate potential biases, a systematic approach was employed to extract the data. Coded patient records were used to ensure confidentiality and minimize selection and reporting bias. By adhering to a clear, predefined set of diagnostic criteria based on pathology reports and the WHO 2017 classification system, classification bias was reduced. Further steps were implemented to reduce additional bias sources. Selection bias was minimized by including all consecutive eligible patients during the study period. Information bias was addressed through double data entry and cross‐checking of extracted variables by two independent reviewers. Measurement bias was reduced by using standardized laboratory procedures across all cases.

### 2.7 Study Size

For the survival analysis, particularly the Cox proportional hazards model, the required sample size was calculated to detect a significant difference in survival outcomes based on the NLR. Assuming a Hazard Ratio (HR) of 0.30 for the low NLR group relative to the high NLR group, an event rate of 60% (the proportion of patients expected to experience death), and a significance level of 0.05 (alpha), with 80% power (beta = 0.2), the sample size was calculated using the following formula:

$$N=\frac{{{(Z}_{\alpha /2}+Z_{\beta })}^{2}}{{\left(\mathrm{HR}-1\right)}^{2}\;\cdot \;p\;\cdot \;(1-p)}$$

Where *Z*_*α*/2_ is the critical value for a two‐tailed test at the 5% significance level (1.96), *Zβ* is the critical value for 80% power (0.84), *HR* is the Hazard Ratio (0.33), and *p* is the event rate (60%). Using the above formula, a total sample size of 66 patients was estimated. Additionally, for logistic regression, considering the number of predictor variables (5 variables: NLR group, tumor grade, metastasis status, age, and gender), the required sample size was estimated using the following rule of thumb:

$$N=\;\frac{10\;\cdot \;k}{p}$$

Where *k* is the number of predictor variables [5], and *p* is the minimum event rate (0.60). This calculation suggests that at least 83 patients would be required for logistic regression analysis. However, due to practical limitations and data availability, a final sample size of 80 patients was selected. This sample size was deemed adequate for both survival analysis and logistic regression, while maintaining sufficient statistical power to detect meaningful associations between the variables of interest and survival outcomes.

### 2.8 Statistical Analysis

Descriptive statistics were used to summarize baseline demographic, clinical, and laboratory characteristics. Continuous variables were expressed as means with standard deviations (SD) or as medians with interquartile ranges (IQR), depending on data distribution, while categorical variables were reported as frequencies and percentages.

For group comparisons, continuous variables were analyzed using the independent samples *t*‐test (for normally distributed data) or the Mann–Whitney *U* test (for non‐normally distributed data). Categorical variables were compared using the *χ* ^2^ test or Fisher's exact test, as appropriate. Longitudinal changes in laboratory parameters were assessed with the Wilcoxon signed‐rank test to evaluate within‐group differences (pre‐ vs. Posttreatment) and the Mann–Whitney *U* test for between‐group comparisons (survivors vs. non‐survivors).

Baseline (“pre”) values were defined as measurements obtained at diagnosis before initiation of treatment. Posttreatment (“post”) assessments were pre‐specified as follows:

- Surgical cases: 3 months after resection, to allow for recovery and to capture early inflammatory changes.
- Chemotherapy cases: After two cycles, in accordance with standardized response assessments based on RECIST guidelines.

This schedule was determined a priori and aligns with previous studies demonstrating that 3‐month intervals are optimal for detecting treatment‐related hematological changes while minimizing bias from acute postoperative inflammation or early chemotherapy toxicity [24, 25].

Survival analyses were pre‐specified and conducted using the Kaplan–Meier method, with differences between groups compared by the log‐rank test. Cox proportional hazards regression was applied to examine associations between candidate variables (e.g., age, metastasis, Ki‐67, inflammatory markers) and overall survival, with hazard ratios (HRs) and 95% confidence intervals (CIs) reported. Logistic regression with a forward stepwise approach was used to identify predictors of vital status, with variables selected a priori if they demonstrated significance in univariate analysis (*p* < 0.05) or were considered clinically relevant (e.g., age, metastasis, Ki‐67). Neuroendocrine neoplasm (NEN) type and tumor grade were excluded from the final multivariate model due to collinearity with Ki‐67 and metastasis (variance inflation factor [VIF]: NEN type = 12.44; grade = 11.47). Subgroup analyses, such as evaluation of baseline NLR in patients with metastatic or unresectable disease, were exploratory in nature.

All *p* values were two‐sided, and a threshold of *p* < 0.05 was defined a priori as statistically significant. Reporting of *P*‐values followed current recommendations (e.g., *p* < 0.001, *p* = 0.01, *p* = 0.34, *p* > 0.99). Statistical analyses were performed using SPSS Statistics version 27 (IBM Corp., Armonk, NY, USA) and R version 4.3.2 (R Foundation for Statistical Computing, Vienna, Austria).

Data completeness was assessed before analysis. Variables with < 5% missing data were analyzed using complete‐case analysis. For variables with > 5% but < 15% missing data, multiple imputation (five iterations using chained equations) was performed. Cases missing outcome data (vital status) were excluded from survival analyses.

### 2.9 Ethical Considerations

The study was conducted in accordance with ethical guidelines for observational research (IR.IUMS.FMD.REC.1399.429) and received approval from the institutional ethics committee. Written informed consent was obtained from all participants for the use of their medical data, and all records were anonymized to maintain patient confidentiality.

## 3 Results

### 3.1 Baseline Characteristics and Their Association With Survival in Gastrointestinal Neuroendocrine Neoplasms (g‐NENs)

The baseline demographic, clinical, and tumor characteristics of 80 patients with g‐NENs were compared by vital status (alive: *n* = 49; deceased: *n* = 31). The mean age was higher in deceased patients (63.8 ± 12.9 years) than in survivors (53.1 ± 12.5 years, *p *< 0.001). Sex distribution was similar between groups (male: 58.1% vs. 49.0%, *p* = 0.57), as was family history of cancer (16.1% vs. 10.2%, *p* = 0.67) and year of diagnosis (2019 vs. 2018: 48.4% vs. 44.9%, *p* = 0.94). Treatment patterns differed significantly: chemotherapy was more frequent in deceased patients (58.1% vs. 12.2%), while surgery was more common in survivors (63.3% vs. 19.4%, *p* < 0.001). Metastatic disease was present in 64.5% of the deceased compared with 30.6% of the survivors (*p* = 0.009). Tumor grade and type also showed strong associations with outcome: Grade 3 tumors were more common among the deceased (80.7% vs. 34.7%), and NEC histology predominated in the deceased group (80.7% vs. 34.7%, both *p* < 0.001). By contrast, chromogranin (64.5% vs. 79.6%, *p* = 0.22) and synaptophysin (96.8% vs. 98.0%, *p* > 0.99) expression did not differ between groups. The Ki‐67 proliferation index was substantially higher in deceased patients (median 50.0%, IQR 12.0–77.5) than in survivors (median 2.0%, IQR 1.0–3.0, *p* < 0.001). A detailed summary of baseline variables is provided in Table 1.

**Table 1:** Baseline demographic, clinical, and tumor characteristics of patients stratified by vital status.

Variable	Levels	Total (*n* = 80)	Vital status		*p* value
			Alive (*n* = 49)	Dead (*n* = 31)	
Sex	Male	42 (52.50)	24 (48.98)	18 (58.06)	0.573
	Female	38 (47.50)	25 (51.02)	13 (41.94)	
Age (years)	—	57.23 ± 13.65	53.08 ± 12.53	63.77 ± 12.93	< 0.001
Diagnosis year	2018	43 (53.75)	27 (55.10)	16 (51.61)	0.940
	2019	37 (46.25)	22 (44.90)	15 (48.39)	
Family history	No	70 (87.50)	44 (89.80)	26 (83.87)	0.665
	Yes	10 (12.50)	5 (10.20)	5 (16.13)	
Treatment	Unknown	14 (17.50)	7 (14.29)	7 (22.58)	< 0.001
	Underwent surgery	37 (46.25)	31 (63.27)	6 (19.35)	
	Received chemotherapy	24 (30.00)	6 (12.24)	18 (58.06)	
	Administered Sandostatin LAR	5 (6.25)	5 (10.20)	0 (0.00)	
Tumor location	Foregut	62 (77.50)	37 (75.51)	25 (80.65)	0.361
	Midgut	11 (13.75)	6 (12.24)	5 (16.13)	
	Hindgut	7 (8.75)	6 (12.24)	1 (3.23)	
Metastasis	Unknown	2 (2.50)	2 (4.08)	0 (0.00)	0.009
	Present	35 (43.75)	15 (30.61)	20 (64.52)	
	Absent	43 (53.75)	32 (65.31)	11 (35.48)	
Chromogranin	Positive	59 (73.75)	39 (79.59)	20 (64.52)	0.218
	Negative	21 (26.25)	10 (20.41)	11 (35.48)	
Synaptophysin	Positive	78 (97.50)	48 (97.96)	30 (96.77)	1.000
	Negative	2 (2.50)	1 (2.04)	1 (3.23)	
NEN type	NET	38 (47.50)	32 (65.31)	6 (19.35)	< 0.001
	NEC	42 (52.50)	17 (34.69)	25 (80.65)	
Grade	Grade 1	30 (37.50)	26 (53.06)	4 (12.90)	< 0.001
	Grade 2	8 (10.00)	6 (12.24)	2 (6.45)	
	Grade 3	42 (52.50)	17 (34.69)	25 (80.65)	
Ki‐67 (%)	—	3.00 (1, 42.50)	2.00 (1, 3)	50.00 (12, 77.50)	< 0.001

*Note:* The baseline characteristics of patients were stratified by vital status and summarized. Continuous variables were described as mean ± standard deviation (SD) or median (interquartile range, IQR), depending on the data distribution. Categorical variables were presented as frequencies and percentages. Comparisons of continuous variables were conducted using the independent samples *t*‐test or Mann–Whitney U test, as appropriate. In contrast, the *χ* ^2^ test or Fisher's exact test was applied for categorical variables.

Abbreviations: CRP, C‐reactive protein; ESR, erythrocyte sedimentation rate; NEC, neuroendocrine carcinoma; NEN, neuroendocrine neoplasm; NET, neuroendocrine tumor; NLR, neutrophil‐to‐lymphocyte ratio; PLR, platelet‐to‐lymphocyte ratio; RNFL, retinal nerve fiber layer.

### 3.2 Longitudinal Changes in Laboratory Parameters and Their Marginal Associations With Vital Status

Longitudinal analysis of laboratory parameters revealed several differences between survivors and non‐survivors. Hemoglobin decreased slightly in both groups, with no significant difference in the magnitude of change (alive: −0.50 g/dL [IQR −1.83 to 0.85] vs. deceased: −0.80 g/dL [IQR −1.70 to −0.20], *p* = 0.69). MCV increased in the deceased group compared with a slight decrease in survivors (1.70 fL [IQR −0.30 to 3.90] vs. −0.45 fL [IQR −2.70 to 1.95], *p* = 0.03). Platelet counts showed modest increases in both groups without significant between‐group differences (alive: +41 [IQR −33 to 92.8] vs. deceased: +14 [IQR −46 to 86], *p* = 0.53). Lymphocyte percentage increased in deceased patients but decreased in survivors (2.90% [IQR −3.75 to 15.7] vs. −2.50% [IQR −16.7 to 7.30], *p* = 0.09). Changes in segment neutrophil percentage were not significant (−0.30% vs. −2.50%, *p* = 0.16). Both NLR and PLR decreased in deceased patients compared with slight increases in survivors, but differences did not reach statistical significance (NLR: −0.85 vs. +0.09, *p* = 0.09; PLR: +5.30 vs. +26.8, *p* = 0.09). Overall, no laboratory parameter demonstrated a statistically significant differential change by survival status after treatment, although MCV showed a modest but significant increase among deceased patients. Detailed results are provided in Table 2.

**Table 2:** Longitudinal changes in laboratory variables stratified by vital status in gastrointestinal neuroendocrine neoplasms.

Laboratory variables	Time point	Vital status		*p* value
		Alive	Dead	
HB (g/dL)	Baseline (pre)	12.50 (10.65, 14.40)	11.60 (10.10, 13.10)	0.126
	3 months posttreatment (post)	12.20 (10.68, 14.10)	10.80 (9.60, 12.80)	0.058
	Post–pre	−0.50 (−1.83, 0.85)	−0.80 (−1.70, −0.20)	0.685
	*p* value	0.244	0.043	—
MCV (fL)	Baseline (pre)	87.00 (84.05, 89.65)	86.10 (78.60, 93.70)	0.726
	3 months posttreatment (post)	86.75 (83.90, 89.93)	86.50 (82.60, 96.60)	1.000
	Post–pre	−0.45 (−2.70, 1.95)	1.70 (−0.30, 3.90)	0.032
	*p* value	0.552	0.021	—
PLT (10^9^/L)	Baseline (pre)	232.00 (186.00, 276.50)	250.00 (180.00, 290.00)	0.518
	3 months posttreatment (post)	251.00 (221.00, 304.00)	260.00 (222.00, 325.00)	0.845
	Post–pre	41.00 (−33.00, 92.75)	14.00 (−46.00, 86.00)	0.530
	*p* value	0.065	0.418	—
WBC (10^9^/L)	Baseline (pre)	8.00 (5.90, 9.20)	8.00 (6.80, 12.00)	0.398
	3 months posttreatment (post)	7.35 (5.70, 9.35)	7.20 (5.70, 10.00)	0.875
	Post–pre	0.75 (−1.25, 2.75)	−1.40 (−2.90, 1.50)	0.158
	*p* value	0.334	0.298	—
Segment (%)	Baseline (pre)	63.30 (55.00, 79.10)	73.60 (70.10, 81.40)	0.007
	3 months posttreatment (post)	65.05 (53.18, 82.70)	69.90 (49.80, 76.30)	0.705
	Post–pre	−0.30 (−12.23, 22.58)	−2.50 (−20.60, 5.40)	0.162
	*p* value	0.610	0.156	—
Lymph (%)	Baseline (pre)	28.40 (14.40, 37.85)	17.70 (10.70, 21.70)	0.001
	3 months posttreatment (post)	28.20 (12.25, 35.30)	22.70 (13.50, 37.70)	0.990
	Post–pre	−2.50 (−16.70, 7.30)	2.90 (−3.75, 15.65)	0.087
	*p* value	0.393	0.048	—
NLR	Baseline (pre)	2.16 (1.45, 5.40)	4.14 (3.20, 7.40)	0.035
	3 months posttreatment (post)	2.33 (1.40, 6.76)	3.11 (1.32, 5.60)	0.638
	Post–pre	0.09 (−1.40, 5.20)	−0.85 (−3.06, 1.03)	0.089
	*p* value	0.478	0.185	—
PLR	Baseline (pre)	143.00 (94.90, 186.50)	187.00 (109.00, 281.00)	0.035
	3 months posttreatment (post)	149.00 (104.75, 292.00)	128.00 (104.00, 211.00)	0.638
	Post–pre	26.80 (−21.55, 145.25)	5.30 (−245.00, 58.00)	0.089
	*p* value	0.036	0.679	—

*Note:* Changes in laboratory variables over time were analyzed and stratified by vital status. Continuous variables were described as median (interquartile range, IQR). The Wilcoxon signed‐rank test was employed to compare paired values (baseline vs. posttreatment) within each group. In contrast, the Mann–Whitney *U* test was applied to assess differences between the “Alive” and “Dead” groups. *p* values were calculated to indicate the statistical significance of these comparisons.

Abbreviations: HB, hemoglobin; MCV, mean corpuscular volume; NLR, neutrophil‐to‐lymphocyte ratio; PLR, platelet‐to‐lymphocyte ratio; PLT, platelet count; WBC, white blood cell count.

### 3.3 Longitudinal Hematological Changes and Inflammatory Patterns in Metastatic and Non‐Metastatic Patients

**Figure 1:**
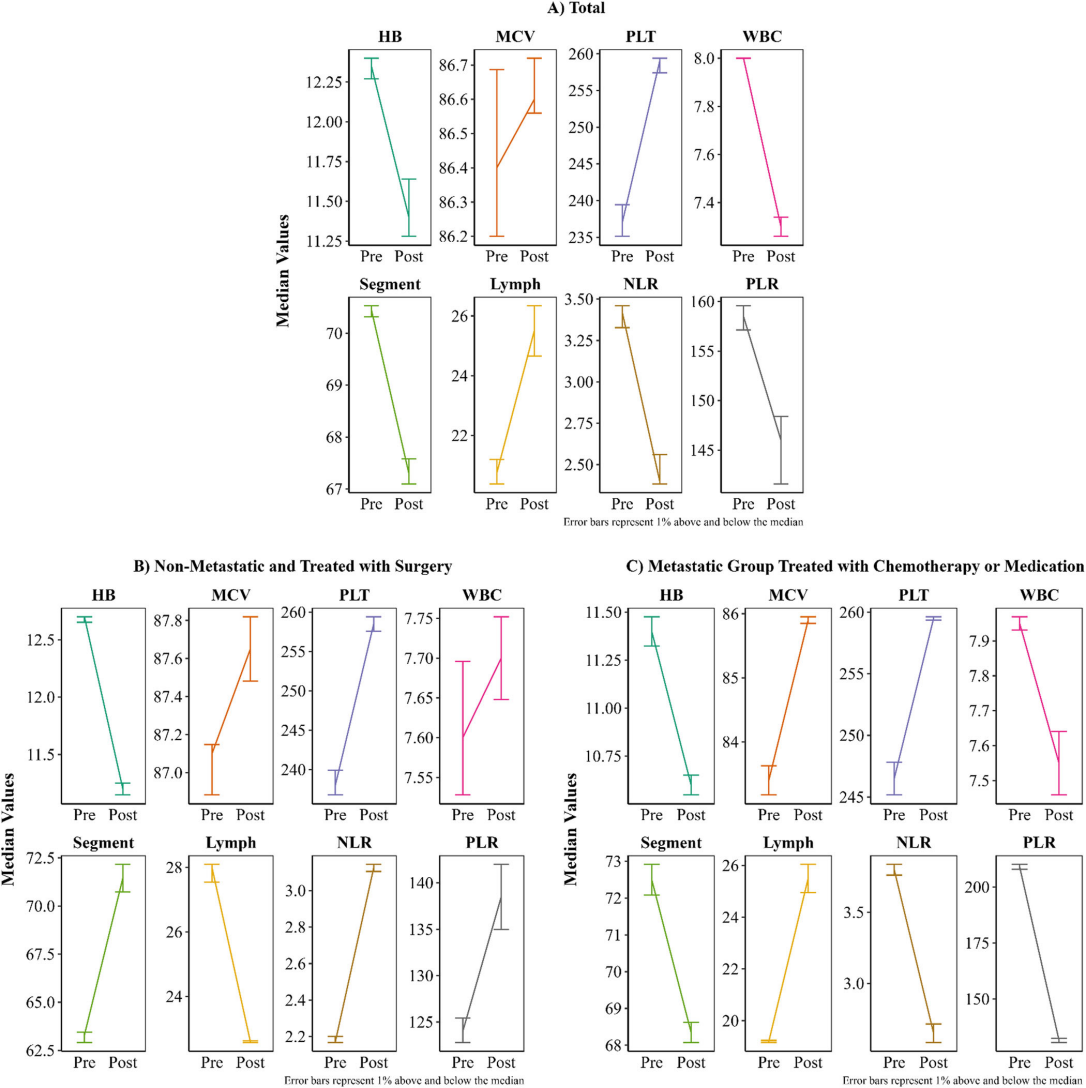
Longitudinal changes in hematological parameters, including neutrophil‐to‐lymphocyte and platelet‐to‐lymphocyte ratios, before and after treatment in gastrointestinal neuroendocrine neoplasms. The figure presents median parameter changes across three groups: (A) the entire cohort, (B) non‐metastatic patients treated with surgery, and (C) metastatic patients treated with chemotherapy or medical therapy.

Figure 1 shows longitudinal changes in median hematological parameters before and after treatment across three groups: the entire cohort (Panel A), non‐metastatic patients undergoing surgery (Panel B), and metastatic patients receiving chemotherapy or medical therapy (Panel C). HB levels decreased after treatment in all groups, with the largest decline observed in the non‐metastatic surgical subgroup. MCV increased consistently across all groups. PLT counts rose in the overall cohort and in non‐metastatic patients but showed minimal change in metastatic patients. WBC decreased in the overall cohort and metastatic patients, while a modest increase was observed in the non‐metastatic subgroup. Segment neutrophil percentage decreased in the overall and metastatic groups but increased in the non‐metastatic subgroup. Lymphocyte percentage decreased in non‐metastatic patients and increased in metastatic patients. NLR and PLR increased in the non‐metastatic subgroup and decreased in metastatic patients.

### 3.4 Logistic Analysis of Factors Influencing Vital Status in Gastrointestinal Neuroendocrine Neoplasms

Univariate logistic regression identified several variables associated with vital status. Older age was linked to higher odds of death (OR 1.07, 95% CI 1.03–1.12, *p* = 0.001), as was the presence of metastasis (OR 3.88, 95% CI 1.49–10.11, *p* = 0.006). Higher Ki‐67 levels (OR 1.05, 95% CI 1.03–1.07, *p* < 0.001), NEC histology (OR 7.84, 95% CI 2.84–24.61, *p* < 0.001), and Grade 3 tumors (OR 9.56, 95% CI 3.07–36.96, *p* < 0.001) were also significantly associated with mortality. Among hematologic parameters, lower lymphocyte counts (OR 0.92, 95% CI 0.88–0.97, *p* = 0.002) and higher baseline segment values (OR 1.06, 95% CI 1.02–1.10, *p* = 0.006) predicted poor outcomes.

In the multivariate stepwise model, age (OR 1.09, 95% CI 1.03–1.16, *p* = 0.006), metastasis (OR 9.10, 95% CI 1.55–53.35, *p* = 0.01), and Ki‐67 (OR 1.04, 95% CI 1.02–1.06, *p *< 0.001) remained independent predictors of mortality. Blood parameters also retained significance: higher segment values (OR 0.80, 95% CI 0.65–0.99, *p* = 0.04) and lower lymphocyte counts (OR 0.74, 95% CI 0.58–0.95, *p* = 0.02) were associated with death after adjustment.

Although NEN type and grade showed strong associations in univariate analysis, they were excluded in the multivariate stepwise model, likely due to collinearity with Ki‐67. Full results of univariate and multivariate analyses are reported in Table 3.

**Table 3:** Univariate and multivariate logistic regression analysis of variables associated with vital status.

Variable	Level	Univariate		Multivariate	
		OR (95% CI)	*p* value	OR (95% CI)	*p* value
Age	—	1.07 (1.03, 1.12)	**0.001**	1.09 (1.03, 1.16)	0.006
Diagnosis year	2019 vs. 2018	1.15 (0.47, 2.85)	0.760		
Sex	Female vs. male	0.69 (0.28, 1.71)	0.429		
Family history	Yes vs. no	1.69 (0.43, 6.63)	0.439		
Treatment	Chemotherapy vs. surgery	15.50 (4.64, 60.58)	< 0.001		
	Administered Sandostatin LAR vs. surgery	0.00 (0.00, Inf)	0.993		
Tumor location	Midgut vs. foregut	1.23 (0.32, 4.53)	0.750		
	Hindgut vs. foregut	0.25 (0.01, 1.57)	0.208		
Metastasis	Present vs. absent	3.88 (1.49, 10.11)	**0.006**	9.10 (1.55, 53.35)	0.014
Ki‐67	—	1.05 (1.03, 1.07)	< **0.001**	1.04 (1.02, 1.06)	< 0.001
Chromogranin	Negative vs. positive	2.14 (0.78, 6.00)	0.139		
Synaptophysin	Negative vs. positive	1.60 (0.06, 41.49)	0.743		
NEN type	NEC vs. NET	7.84 (2.84, 24.61)	< **0.001**		
Grade	Grade 2 vs. grade 1	2.17 (0.26, 14.20)	0.429		
	Grade 3 vs. grade 1	9.56 (3.07, 36.96)	< 0.001		
HB (baseline)	—	0.86 (0.70, 1.04)	0.122		
MCV (baseline)	—	0.99 (0.93, 1.04)	0.600		
PLT (baseline)	—	1.00 (1.00, 1.01)	0.770		
WBC (baseline)	—	1.11 (0.96, 1.29)	0.177		
Segment (baseline)	—	1.06 (1.02, 1.10)	**0.006**	0.80 (0.65, 0.99)	0.043
Lymph (baseline)	—	0.92 (0.88, 0.97)	**0.002**	0.74 (0.58, 0.95)	0.017
NLR (baseline)	—	1.11 (0.98, 1.27)	0.105		
PLR (baseline)	—	1.01 (1.00, 1.01)	**0.039**		
HB (3 months posttreatment)	—	0.77 (0.55, 1.03)	0.090		
MCV (3 months posttreatment)	—	1.01 (0.94, 1.10)	0.767		
PLT (3 months posttreatment)	—	1.00 (0.99, 1.00)	0.452		
WBC (3 months posttreatment)	—	0.98 (0.76, 1.25)	0.858		
Segment (3 months posttreatment)	—	0.99 (0.96, 1.03)	0.653		
Lymph (3 months posttreatment)	—	1.01 (0.98, 1.06)	0.482		
NLR (3 months posttreatment)	—	0.95 (0.80, 1.09)	0.480		
PLR (3 months posttreatment)	—	1.00 (0.99, 1.00)	0.410		
HB (post–pre)	—	0.92 (0.68, 1.21)	0.545		
MCV (post–pre)	—	1.18 (1.01, 1.46)	0.083		
PLT (post–pre)	—	1.00 (0.99, 1.00)	0.245		
WBC (post–pre)	—	0.85 (0.66, 1.06)	0.163		
Segment (post–pre)	—	0.97 (0.93, 1.00)	0.088		
Lymph (post–pre)	—	1.05 (1.00, 1.11)	0.068		
NLR (post–pre)	—	0.87 (0.72, 1.00)	0.078		
PLR (post–pre)	—	1.00 (0.99, 1.00)	0.059		

*Note:* Univariate and multivariate logistic regression analyses using a forward stepwise approach were conducted to identify variables associated with vital status. Odds ratios (ORs), 95% confidence intervals (CIs), and *p* values were used to evaluate the strength and significance of associations. Univariate analysis examined individual variables, while multivariate analysis adjusted for potential confounders to determine their independent effects. The forward stepwise method enabled the sequential inclusion of variables based on statistical significance, ensuring that the most relevant predictors were retained in the final model. Logistic regression was employed to calculate ORs and assess associations. Bold values indicate statistical significance (*p* < 0.05).

Abbreviations: CI, confidence interval; NEC, neuroendocrine carcinoma; NEN, neuroendocrine neoplasm; NET, neuroendocrine tumor; OR, odds ratio.

### 3.5 Survival Analysis and Prognostic Significance of NLR in Gastrointestinal Neuroendocrine Neoplasms

**Figure 2:**
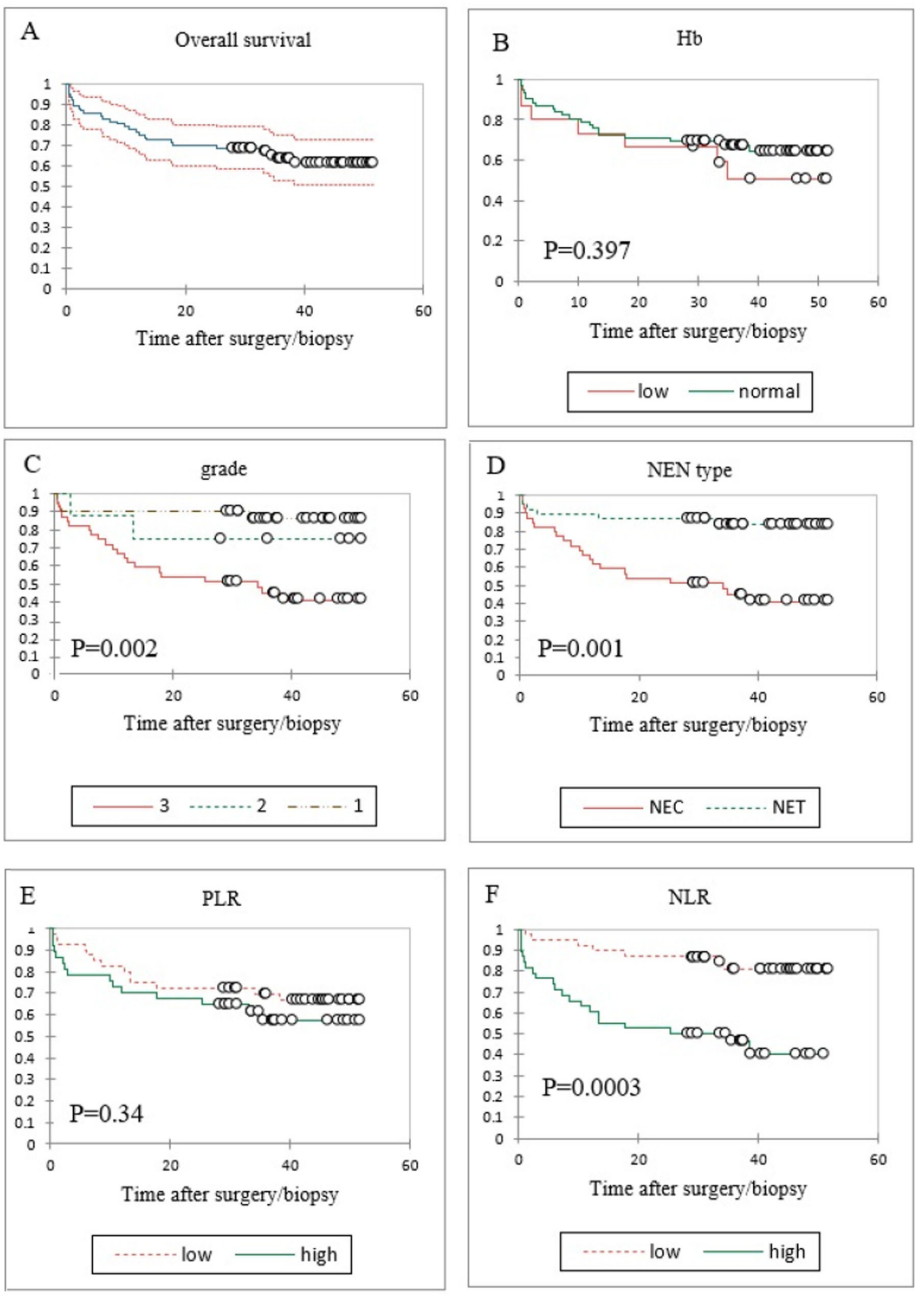
Kaplan–Meier survival analysis with 95% confidence intervals: (A) overall survival; (B) survival stratified by hemoglobin levels (≥ 12 vs. < 12); (C) Comparison of survival across different NEN grades; (D) Survival in NEC vs. NET; (E) Survival based on PLR (≥ 159 vs. < 159); (F) Survival based on NLR (≥ 3.4 vs. < 3.4). Abbreviations: NEN, neuroendocrine neoplasm; NET, neuroendocrine tumor; NLR, neutrophil‐to‐lymphocyte ratio; PLR, platelet‐to‐lymphocyte ratio.

Of the 80 patients, 77 were included in the survival analysis after excluding three with incomplete death dates (Figure 2). The mean overall survival time for the cohort was 36.4 months (SE 2.4), with an average follow‐up of approximately 3 years. The median overall survival was not reached in the total cohort; however, patients with NEC had a median survival of 34.2 months. Survival differed significantly across tumor grades (log‐rank *p* = 0.002) and NEN type (NET vs. NEC, log‐rank *p* = 0.001), but not by hemoglobin level (< 12 vs. ≥ 12 g/dL, log‐rank *p* = 0.40) or tumor site.

The prognostic analysis of inflammatory markers demonstrated that patients with low baseline NLR (< 3.4) had significantly better survival compared with those with high NLR (≥ 3.4) (log‐rank *χ*² = 12.88, *p* < 0.001; Figure 2F). In contrast, PLR groups defined by the cohort median (159) showed no significant differences in survival (log‐rank *p* = 0.34; Figure 2E). In Cox regression analysis, NLR remained independently associated with survival after adjusting for tumor grade and metastasis (global *χ*² = 24.39, df = 4, *p* < 0.001).

To further assess the prognostic relevance of NLR in advanced disease, we analyzed baseline NLR values in patients with metastatic or unresectable tumors (*n* = 24). Among this subgroup, baseline NLR did not differ significantly between survivors (mean 5.91, SD 5.70) and non‐survivors (mean 5.68, SD 3.48; *p* = 0.91) (Table 4). These findings suggest that, while NLR is a significant prognostic biomarker in the overall g‐NEN population, its predictive utility is limited in patients with metastatic or unresectable disease.

**Table 4:** Independent samples *t*‐test results for baseline NLR and vital status in patients with metastatic/unresectable gastrointestinal neuroendocrine neoplasms.

	Vital status	*N*	Mean	SD	*p* value
NLR	Alive	6	5.91	5.70	0.91
	Dead	18	5.68	3.48	

## 4 Discussion

This study provides novel insights into the prognostic significance of inflammatory markers and histopathological features in gastrointestinal neuroendocrine neoplasms (g‐NENs). By evaluating survival outcomes in relation to NLR, PLR, tumor grade, and metastasis status, our findings suggest that NLR may serve as a meaningful prognostic indicator in g‐NENs, whereas baseline NLR did not retain predictive value in patients with metastatic or unresectable disease. In contrast, PLR showed no independent prognostic significance in our cohort, despite reports of prognostic utility in other malignancies such as colorectal, NSCLC, and cholangiocarcinoma. These differences may reflect sample size limitations, cutoff variability across studies, or treatment‐related effects that attenuate PLR's prognostic signal in this setting.

Our findings indicate that patients with a low NLR (< 3.4) exhibited significantly improved survival outcomes compared to those with a high NLR (≥ 3.4). Specifically, the odds of 3‐year survival in the low NLR group were nearly double those of the high NLR group. This trend was supported by Cox regression analysis, which indicated that the hazard of death in the low NLR group was approximately one‐third of that in the high NLR group, even after adjusting for metastasis and tumor grade.

The 3‐month postsurgical interval was selected to capture the resolution of acute inflammation while avoiding confounding by delayed recurrence. For chemotherapy, response assessments after two cycles align with RECIST guidelines. This timing balances early detection of treatment effects with clinical practicality, as shorter intervals may reflect transient inflammation rather than true prognostic shifts

Although these results appear consistent with prior reports on the prognostic utility of NLR, the small sample size and retrospective design of our study warrant cautious interpretation [26, 27]. In a survey by de Lima et al. involving 96 patients with advanced‐stage NETs, NLR was identified as a significant prognostic biomarker in patients undergoing PRRT, where low NLR (< 1.8) was associated with significantly longer overall survival (77.5 months vs. 47.7 months), underscoring its reproducible value across clinical contexts [28]. By contrast, although some prior studies have suggested that PLR may serve as a prognostic marker in gastrointestinal NETs [29]. Our findings did not demonstrate an independent association, highlighting the need for cautious interpretation of PLR compared with the more consistent prognostic role observed for NLR. Notably, Giannetta et al. [30] provided a comprehensive overview of systemic inflammatory response markers in NENs, reinforcing their potential clinical value while cautioning against overinterpretation. Their review highlighted that indices such as NLR and PLR capture the interplay between host immunity and tumor biology, but their prognostic reliability is hindered by heterogeneous cutoffs, differences in tumor stage, and variability in treatment regimens. Importantly, the authors advocated for integrating these markers into multiparametric models (alongside histopathological and molecular features) to enhance patient stratification and therapeutic decision‐making. These insights are consistent with our findings and emphasize the need for standardized thresholds and prospective validation before routine incorporation of inflammatory biomarkers into clinical practice.

Furthermore, higher tumor grades (Grade 3) and increased Ki‐67 proliferation indices were significantly correlated with poorer survival outcomes, consistent with previous reports in the literature [12, 31]. These findings align with the study by Barnes et al. [32], who investigated the relationship between Ki‐67 proliferation indices and neuroendocrine tumors. In their analysis of 36 patients who underwent both tissue sampling and tumor resection, elevated Ki‐67 levels were strongly linked to more aggressive tumor behavior and reduced survival rates [32]. Similarly, Shi et al. [14] analyzed 514 patients with GEP‐NENs and concluded that Ki‐67 variability was associated with a worse prognosis, as patients exhibiting such variability had significantly shorter overall survival [14].

As anticipated, the presence of metastatic disease significantly influenced survival outcomes, with patients exhibiting metastases experiencing poorer prognoses compared to those with localized disease. Notably, Ki‐67 levels were markedly higher in deceased patients, corroborating its role as a critical marker of disease aggressiveness and metastatic potential. Although PLR was associated with death status in our study, the difference between the high and low PLR groups did not reach statistical significance. This contrasts with findings from other studies on g‐NENs, where PLR was identified as a predictor of disease progression and poor survival [29, 33], and aligns with the findings of Kulahci and Koseci [34], who observed no significant relationship between PLR and histopathological features in neuroendocrine tumors. On the other hand, they emphasized that an elevated NLR (above 3.01) was significantly associated with high tumor grade, increased Ki‐67 proliferation index, distant metastasis, and lymphovascular invasion, highlighting the potential of NLR as an independent predictive biomarker in NETs [34]. This discrepancy may be attributable to differences in study populations, underlying tumor biology, or the limited sample size of our cohort. Chronic inflammation plays a central and multifaceted role in the initiation and progression of neuroendocrine tumors (NETs) and other malignancies, with its impact modulated by the predominance and functional characteristics of specific immune cell subsets [35, 36, 37].

Contrary to prior studies, NLR did not predict survival in metastatic/nonsurgical cases (*p* = 0.907), possibly due to the limited sample size or unique inflammatory dynamics in advanced gastric neoplasms. This contrasts with reports in resectable tumors, highlighting the need for context‐specific validation of biomarkers. Future studies should investigate NLR's role in larger, homogeneous cohorts of unresectable disease.

In gastroenteropancreatic NETs, prolonged inflammation can hyperstimulate enteroendocrine cells, potentially triggering hyperplasia, neoplastic transformation, and tumor growth [36, 38]. These tumors are highly vascularized and express growth factors, pro‐inflammatory cytokines, and tyrosine kinase receptors, all of which contribute to their pathogenesis [39, 40]. Elevated levels of inflammatory markers, such as CRP, vascular endothelial growth factor (VEGF), and interleukins, have been linked to poor outcomes in various solid tumors, including NETs [40, 41, 42].

Among inflammation‐based markers, the NLR and PLR have gained recognition for their prognostic significance in assessing tumor progression [41, 42, 43, 44, 45, 46]. NLR, in particular, has shown robust prognostic value in a range of solid tumors, including NETs [47, 48, 49, 50, 51]. Elevated NLR reflects a pro‐inflammatory state dominated by neutrophils, which release reactive oxygen species and cytokines that promote DNA damage, tumor growth, and immune evasion. Concurrently, lymphopenia signifies impaired adaptive immune responses, further enabling tumor progression. Platelets also contribute by shielding circulating tumor cells from immune‐mediated destruction and facilitating metastasis through angiogenesis and endothelial adhesion [35, 41]. The interplay between systemic inflammation and tumor dynamics highlights the potential utility of markers such as NLR and, to a lesser extent, PLR as promising but unconfirmed prognostic tools.

Interestingly, our analysis did not find significant associations between CRP or erythrocyte sedimentation rate (ESR) and survival outcomes, nor with tumor grade or Ki‐67 index. This diverges from studies in pancreatic g‐NENs and other cancers, where elevated acute‐phase proteins, such as CRP, have been linked to poor prognosis [17, 52]. The lack of association in our study could be due to the small sample size, limited variability in CRP levels, or the unique tumor biology of g‐NENs.

The present study observed that NLR did not demonstrate prognostic value in patients with unresectable or metastatic gastric neoplasms (g‐NENs); however, this finding requires careful contextualization. Several factors may explain this result. First, the relatively small sample size in the metastatic subgroup may have limited the statistical power to detect meaningful differences. In addition, advanced tumors are characterized by marked biological heterogeneity, where systemic inflammation, tumor‐driven immunosuppression, and metabolic derangements often overshadow baseline immune activity. The heterogeneity of treatment regimens, particularly the varying chemotherapy protocols administered in this population, further introduces confounding effects that complicate the interpretation of NLR. Importantly, chemotherapy itself induces bone marrow suppression and lymphopenia, which may obscure the prognostic relevance of NLR by artificially altering leukocyte counts [53, 54].

Beyond treatment effects, the unique inflammatory dynamics of advanced‐stage g‐NENs may differ substantially from earlier disease, potentially diminishing the predictive value of a single baseline NLR measurement. Evidence from large umbrella reviews has consistently linked elevated NLR to poor outcomes across solid tumors, yet these same analyses underscore that its prognostic strength is strongly dependent on cancer stage, treatment context, and methodological design. Indeed, studies in advanced gastrointestinal malignancies suggest that dynamic changes in NLR during therapy may be more informative than baseline values [54]. Moreover, additional factors not captured in this analysis (including specific genetic mutations, molecular signatures, or variations in the tumor microenvironment) may exert a more dominant influence on survival outcomes in advanced disease. Taken together, the lack of prognostic significance in this cohort likely reflects the convergence of limited sample size, treatment‐induced immunosuppression, heterogeneous regimens, and the inherently aggressive biology of metastatic disease. These findings highlight the need for comprehensive, multi‐center investigations that incorporate longitudinal NLR assessments and account for treatment‐related effects, genetic determinants, and microenvironmental influences to more precisely define the prognostic role of NLR in advanced g‐NENs.

Beyond generic systemic inflammation, accumulating evidence suggests that gut microbiota‐driven immune activation may be an important upstream determinant of NLR and PLR in GEP‐NENs. A Mendelian randomization study has reported putative causal links between specific gut microbial taxa, immune cell subsets, and the risk of pancreatic and other GEP‐NENs, supporting a microbiota–immunity–tumor axis [55]. In parallel, a pilot tissue‐based study has demonstrated bacterial infiltration within intestinal and pancreatic NENs, with enrichment of intratumoral microbiota compared with adjacent non‐tumoral tissue. Dysbiosis, disruption of the intestinal barrier, and activation of pattern‐recognition receptors such as TLR4 can trigger downstream NF‐κB and NLRP3 inflammasome signaling, fostering chronic systemic inflammation, cytokine release, and a shift toward neutrophilia with relative lymphopenia. These processes could, at least in part, underlie elevated NLR and PLR and their association with adverse outcomes in g‐NENs. Although microbiome composition and barrier integrity were not assessed in our cohort, gut microbiota–driven inflammation likely represents an important, currently unmeasured source of variability in NLR/PLR and warrants dedicated mechanistic and translational investigation in future studies [56].

This study has several limitations that should be acknowledged. First, its retrospective, single‐center design and relatively small sample size (80 patients, with 31 events) may limit statistical power and generalizability, particularly in subgroup analyses such as metastatic versus non‐metastatic disease. Second, the number of events constrained the complexity of multivariable modeling; including more than three predictors would have increased the risk of overfitting and multicollinearity, as emphasized in Agresti's *An Introduction to Categorical Data Analysis* (Chapter 5, p. 22) [57], and thus we employed a stepwise approach to retain only the most informative variables. Third, our data set did not include validated indicators of nutritional status, such as body mass index, serum albumin, or performance scores. As malnutrition and sarcopenia are common in cancer patients and can markedly influence lymphocyte counts and systemic inflammatory markers, their absence may have confounded the interpretation of NLR and PLR. Fourth, we also lacked data on metabolic syndrome and related conditions, including type 2 diabetes, obesity, fasting glucose, and lipid profiles. These comorbidities are known to modulate systemic inflammation and may independently affect survival outcomes in g‐NENs, representing another source of residual confounding. In addition, emerging evidence suggests that gut microbiota–related mechanisms, including dysbiosis, microbial translocation, and activation of inflammatory pathways such as the TLR4/NF‐κB/NLRP3 axis, may influence systemic immune responses and thereby affect NLR and PLR values. Because microbiome composition and gut barrier integrity were not assessed in our data set, these biologically relevant factors represent an additional unmeasured confounder that could contribute to variability in inflammation‐based biomarkers. Finally, heterogeneity in treatment modalities and incomplete follow‐up for some patients further limit the scope of inference. Future multicenter, prospective studies incorporating nutritional and metabolic assessments, standardized treatment data, and larger patient cohorts are warranted to validate and extend our findings.

Further prospective studies are warranted to validate these findings and to determine whether inflammatory biomarkers can be reliably integrated into clinical assessment models for improved risk stratification. The observed association between Ki‐67 and PLR also underscores the need for further investigation into their potential as prognostic indicators for survival. Although this study employed robust statistical frameworks and transparent methodologies to ensure the reliability of results, larger multi‐center cohorts are essential to confirm the generalizability of these biomarkers across diverse populations. Future research should also incorporate death certificate data or cancer registry linkages to ascertain cause‐specific mortality accurately.

## 5 Conclusion

This study suggests that the NLR may serve as an accessible and cost‐effective prognostic indicator in patients with g‐NENs, with a lower NLR (< 3.4) associated with improved survival after adjustment for tumor grade and metastasis. Although PLR values were higher among deceased patients, this relationship did not reach statistical significance. Ki‐67 index and tumor grade remained robust predictors of poor prognosis, particularly in grade 3 tumors. Notably, baseline NLR did not show prognostic value in metastatic or unresectable disease, suggesting a potential stage‐specific role. These findings, if validated in larger prospective cohorts, may support the integration of simple inflammatory biomarkers such as NLR into current risk‐stratification models alongside histopathological parameters to guide individualized follow‐up and management strategies in g‐NENs. Given the retrospective design and limited sample size, the present results should be interpreted as hypothesis‐generating and warrant confirmation in multicenter studies.

## Author Contributions

**Hoda Mahdavi:** conceptualization, supervision, writing original draft, writing, review, and editing, project administration. **Vahid Kaveh:** conceptualization, methodology, writing original draft, writing, review, and editing. **Mahdiye Ijadi Nasrabadi:** data curation, writing, review, and editing. **Mohammad Amin Karimi:** methodology, data curation, writing, review, and editing. **Mehdi Azizmohammad Looha:** data curation, writing original draft, writing, review, and editing.

## Ethics Statement

This study was conducted in accordance with the ethical principles for human research and was approved by the Ethics Committee of Iran University of Medical Sciences, Faculty of Medicine (Approval ID: IR.IUMS.FMD.REC.1399.429). All procedures were performed in compliance with institutional guidelines and national regulations on human subject research.

## Consent

Written informed consent was obtained from all participants, or from their legal representatives when applicable, before data collection. Participation was voluntary, and confidentiality of all personal information was strictly maintained throughout the study.

## Conflicts of Interest

The authors declare no conflicts of interest.

## Transparency Statement

The lead author, Vahid Kaveh, affirms that this manuscript is an honest, accurate, and transparent account of the study being reported, that no important aspects of the study have been omitted; and that any discrepancies from the study as planned (and, if relevant, registered) have been explained.

## Data Availability

The authors confirm that the data supporting the findings of this study are available within the article. Additional data sets used and/or analyzed during the current study are available from the corresponding author upon reasonable request.
